# Supplementary material for: Automatic comprehensive radiological reports for clinical acute stroke MRIs
Source: Commun Med (Lond). 2023 Jul 10;3:95. doi: 10.1038/s43856-023-00327-4 (PMC10333348; doi:10.1038/s43856-023-00327-4)
Supplement: Supplementary file 1 — Description of Additional Supplementary Files [file 43856_2023_327_MOESM1_ESM.pdf]

Description of Additional Supplementary Files

File Name: Supplementary Data 1

Description: Hyperparameters for the ML models for automatic prediction of infarct location.

File Name: Supplementary Data 2

Description: Performance of the ML models to predict infarct location and hydrocephalus in the training set (n = 1414), in the Cross validation

File Name: Supplementary Data 3

Description: Hyperparameters of the ML models for automatic prediction of infarct location the testing set (n = 464), using automatic lesion segmentation from ADS pipeline

File Name: Supplementary Data 4

Description: Performances ML models to predict infarct location and hydrocephalus in the training set (n = 1414), during cross validation, using automatic lesion segmentation from ADS

File Name: Supplementary Data 5

Description: Performances ML models to predict infarct location and hydrocephalus in the training set (n = 1414), during cross validation, using non-linear brain normalization

File Name: Supplementary Data 6

Description: Source data for this manuscript
