# Supplementary material for: Automatic comprehensive radiological reports for clinical acute stroke MRIs
Source: Commun Med (Lond). 2023 Jul 10;3:95. doi: 10.1038/s43856-023-00327-4 (PMC10333348; doi:10.1038/s43856-023-00327-4)
Supplement: Supplementary file 2 — Supplementary Information [file 43856_2023_327_MOESM2_ESM.pdf]

AUTOMATIC COMPREHENSIVE RADIOLOGICAL REPORTS IN CLINICAL ACUTE STROKE MRIs: Supplementary Information

SUPPLEMENTARY METHODS

Quality Control Index (QCI)

Although efficient on matching the global brain shape, the linear transformation does not match the shape of internal structures. This is a particular issue in elderly people (who often have enlarged ventricles) and in brains with focal lesions. The γ_{OLVR} and γ_{ILVR} were used to create an "internal" QCI, which indicates the degree of match between the template and the subject LV. Furthermore, to detect drastic errors in brain mapping, we estimated the "external" QCI. Similar to the ventricular metrics, two five-voxel-width strips were created inside (ISB) and outside (OSB) the brain mask surface of template. Each strip was subdivided into 24 subregions of similar volume, to provide more precise regional information. The metrics used to calculate the "external" QCI were:

1. γ_{OSBR}: the ratio of the number of the brain-mask voxels in OSB, over the number of voxels in OSB. High γ_{OSBR} indicates large number of brain voxels outside the standardized brain contour.
2. γ_{ISBR}: the ratio of the number of the non-brain-mask voxels in ISB over the number of voxels in ISB. High γ_{ISBR} indicates large number of background voxels inside the standardized brain contour.

The average γ_{OSBR} was 0.2042 ± 0.0628 (medium=0.1929, range [0.0723, 0.5070]). The average γ_{ISBR} was 0.2683 ± 0.0426 (medium=0.2666, range [0.1560, 0.4273]). This means that a small minority of brain voxels did not match the template brain contour, when considering a stringent bandwidth of 5 voxels. Importantly, the small ratio of error was stable over magnetic field and scan manufacturer, as detailed in our publication [1]

The individual "internal" and "external" QCIs were then calculated as the average of γ_{OLVR} and the γ_{ILVR}, and γ_{OSBR} and γ_{ISBR}, respectively, in all the subregions. The population "external" QCI

was computed by averaging the QCIs over the whole sample, while the population "internal" QCI was computed only with the brains in which hydrocephalus or midbrain abnormalities were not visually detected. These indices were added to the radiological reports, as an estimation of the quality of the brain deformation, and consequently, of the extracted QFVs. If the subject QCI is much lower than the the population QCI, our system offers the option of recalculating the QFVs using non-linear brain mapping (implemented with Dipy), which costs extra 3 minutes of processing time.

Measures of model performance and system implementation

Let TP, FP, TN, and FN denote True Positives, False Positives, True Negatives, and False Negatives, respectively.

1. Balanced accuracy (BACC) [2]: to avoid inflated performance issue due to imbalanced classes, BACC is used instead of accuracy.

$$\text{Balanced Accuracy (BACC)} = \frac{1}{2}\left(\frac{\text{TP}}{\text{TP} + \text{FP}} + \frac{\text{TP}}{\text{TP} + \text{FN}}\right) \tag{1}$$

2. Precision: a metric to evaluate how accurate a model's positive predict is true.

$$\text{Precision} = \frac{\text{TP}}{\text{TP} + \text{FP}} \tag{2}$$

3. Sensitivity: a metric to evaluate how the model's ability to detect the positive cases among dataset.

$$\text{Sensitivity} = \frac{\text{TP}}{\text{TP} + \text{FN}} \tag{3}$$

4. F1 score: the harmonic mean of the precision and sensitivity.

$$\text{F1 Score} = \frac{2}{\text{SEN}^{-1} + \text{PPV}^{-1}} \tag{4}$$

5. Cohen's Kappa coefficient (κ) [3]: a statistic to measure the agreement between annotators. Besides evaluating our inter-annotator performance, we also like to consider ML models as extra annotators

and evaluate their performance in the test set, completely hold-out from training.

$$K = \frac{p_o - p_e}{1 - p_e} \quad (5)$$

where p_o is the empirical probability of agreement among raters, and p_e is the expected agreement between random raters. p_e is estimated via the empirical prior probability of each class of raters.

The statistical significance testing was performed by Anova test for continuous data, and by Chi-squared test for categorical data. The metrics of model performance were implemented by scikit-learning module. All the evaluated methods and models were built with TensorFlow[4] (tensorflow-gpu version is 2.0.0) and Keras[5] (2.3.1) framework on Python 3.6. Imaging processing and analysis were built with Nibabel[6], Scipy[7], Dipy[8], Scikit-learning, Scikit-image[9, 10], and SHAP[11]. The experiments were done on a machine with an Intel Core (Intel(R) Xeon(R) CPU E5-2620 v4 @ 2.10GHz) with 2 NVIDIA TITAN XP GPUs (with CUDA 10.1).

SUPPLEMENTARY DATA

Supplementary Data 1. Hyperparameters for the ML models for automatic prediction of infarct location.

Supplementary Data 2. Performance of the ML models to predict infarct location and hydrocephalus in the training set (n = 1414), in the Cross validation.

Supplementary Data 3. Hyperparameters of the ML models for automatic prediction of infarct location the testing set (n = 464), using automatic lesion segmentation from ADS pipeline.

Supplementary Data 4. Performances ML models to predict infarct location and hydrocephalus in the training set (n = 1414), during cross validation, using automatic lesion segmentation from ADS.

Supplementary Data 5. Performances ML models to predict infarct location and hydrocephalus in the training set (n = 1414), during cross validation, using non-linear brain normalization.

SUPPLEMENTARY TABLES

RF MDI importance									
**ROI**	**lesion volume log ml**	**QFV_ACA**	**QFV_MCA**	**QFV_PCA**	**QFV_cerebellar**	**QFV_basilar**	**QFV_LLS**	**QFV_ChThp**	**QFV_watershed**
**ACA**	0.114	0.331	0.242	0.048	0.019	0.031	0.055	0.021	0.139
**MCA**	0.111	0.105	0.430	0.052	0.030	0.029	0.059	0.070	0.114
**PCA**	0.091	0.042	0.102	0.416	0.133	0.022	0.047	0.062	0.085
**cerebellar**	0.046	0.013	0.037	0.091	0.624	0.092	0.016	0.035	0.045
**basilar**	0.073	0.001	0.003	0.018	0.227	0.596	0.002	0.077	0.003
**LatLenticulostriate**	0.068	0.017	0.196	0.023	0.008	0.010	0.618	0.039	0.021
**Choroidal& Thalamoperforating**	0.092	0.019	0.038	0.024	0.055	0.095	0.278	0.369	0.029

RF permutation importance in the training set									
**ROI**	**lesion volume log ml**	**QFV_ACA**	**QFV_MCA**	**QFV_PCA**	**QFV_cerebellar**	**QFV_basilar**	**QFV_LLS**	**QFV_ChThp**	**QFV_watershed**
**ACA**	0.073	0.503	0.139	0.029	0.014	0.026	0.045	0.008	0.102
**MCA**	0.058	0.026	0.461	0.027	0.003	0.003	0.035	0.036	0.023
**PCA**	0.045	0.021	0.055	0.456	0.057	0.004	0.025	0.009	0.023
**cerebellar**	0.007	0.008	0.010	0.016	0.480	0.021	0.007	0.003	0.005
**basilar**	0.025	0.000	0.000	0.004	0.042	0.434	0.005	0.131	0.003
**LatLenticulostriate**	0.022	0.008	0.151	0.008	0.002	0.003	0.427	0.013	0.005
**Choroidal& Thalamoperforating**	0.023	0.004	0.012	0.009	0.032	0.034	0.122	0.302	0.004

RF permutation importance in the testing set									
**ROI**	**lesion volume log ml**	**QFV_ACA**	**QFV_MCA**	**QFV_PCA**	**QFV_cerebellar**	**QFV_basilar**	**QFV_LLS**	**QFV_ChThp**	**QFV_watershed**
**ACA**	-0.074	0.245	-0.017	0.004	0.000	0.000	-0.004	0.000	-0.019
**MCA**	0.044	0.005	0.390	0.032	-0.001	0.000	0.031	0.021	0.005
**PCA**	-0.002	-0.001	0.023	0.346	0.011	-0.008	0.000	0.008	0.006
**cerebellar**	0.002	0.011	-0.001	0.015	0.444	0.010	0.003	0.005	0.005
**basilar**	0.027	0.002	0.000	0.011	-0.029	0.349	0.000	0.139	0.000
**LatLenticulostriate**	-0.002	0.001	0.076	-0.008	0.006	0.003	0.372	0.011	-0.007
**Choroidal& Thalamoperforating**	0.003	-0.002	0.010	0.002	0.019	0.027	0.107	0.209	0.006

Supplementary Table 1. Feature importance as revealed by the analysis of Mean Decrease in Impurity (MDI) and permutation test of the Random Forest (RF) model for arterial territories.

RF MDI importance

ROI	lesion volume log ml	QFV_basal ganglia	QFV_deep white matter	QFV_cerebellum	QFV_frontal	QFV_insula	QFV_internal capsule	QFV_brainstem	QFV_occipital	QFV_parietal	QFV_temporal	QFV_thalamus
**basal ganglia**	0.074	0.693	0.025	0.003	0.019	0.053	0.038	0.015	0.010	0.018	0.036	0.016
**deep white matter**	0.048	0.023	0.613	0.008	0.071	0.019	0.035	0.011	0.022	0.115	0.023	0.014
**cerebellum**	0.063	0.011	0.032	0.597	0.028	0.013	0.022	0.071	0.070	0.027	0.039	0.027
**frontal**	0.024	0.015	0.047	0.001	0.835	0.020	0.015	0.001	0.005	0.024	0.005	0.008
**insula**	0.008	0.047	0.015	0.001	0.035	0.807	0.040	0.001	0.004	0.023	0.016	0.004
**internal capsule**	0.295	0.025	0.032	0.002	0.108	0.040	0.387	0.008	0.007	0.035	0.011	0.051
**brainstem**	0.179	0.002	0.001	0.022	0.001	0.001	0.021	0.739	0.002	0.001	0.001	0.032
**occipital**	0.111	0.028	0.057	0.101	0.056	0.036	0.032	0.027	0.352	0.082	0.069	0.049
**parietal**	0.029	0.004	0.010	0.029	0.041	0.005	0.003	0.002	0.123	0.744	0.006	0.004
**temporal**	0.055	0.019	0.032	0.019	0.068	0.048	0.027	0.012	0.057	0.040	0.606	0.017
**thalamus**	0.065	0.173	0.025	0.029	0.008	0.010	0.105	0.022	0.014	0.009	0.025	0.516

RF permutation importance in the training set

ROI	lesion volume log ml	QFV_basal ganglia	QFV_deep white matter	QFV_cerebellum	QFV_frontal	QFV_insula	QFV_internal capsule	QFV_brainstem	QFV_occipital	QFV_parietal	QFV_temporal	QFV_thalamus
**basal ganglia**	0.039	0.460	0.018	0.001	0.008	0.038	0.019	0.005	0.005	0.008	0.021	0.009
**deep white matter**	0.017	0.002	0.372	0.002	0.049	0.005	0.016	0.003	0.016	0.099	0.005	0.005
**cerebellum**	0.012	0.003	0.012	0.481	0.011	0.005	0.006	0.012	0.016	0.009	0.007	0.007
**frontal**	0.009	0.001	0.019	0.003	0.413	0.009	0.002	0.000	0.000	0.008	0.003	0.001
**insula**	0.002	0.018	0.001	0.000	0.007	0.465	0.003	0.000	0.000	0.007	0.005	0.000
**internal capsule**	0.139	0.010	0.013	0.000	0.092	0.020	0.236	0.005	0.000	0.005	0.002	0.011
**brainstem**	0.138	0.000	0.000	0.015	0.000	0.000	0.007	0.452	0.001	0.000	0.000	0.006
**occipital**	0.058	0.008	0.030	0.036	0.033	0.020	0.012	0.005	0.427	0.043	0.038	0.011
**parietal**	0.009	0.000	0.001	0.007	0.016	0.000	0.000	0.000	0.082	0.341	-0.001	0.001
**temporal**	0.046	0.012	0.017	0.008	0.105	0.025	0.018	0.004	0.043	0.025	0.463	0.009
**thalamus**	0.020	0.039	0.003	0.017	0.000	0.004	0.053	0.011	0.002	0.001	0.011	0.437

RF permutation importance in the testing set

ROI	lesion volume log ml	QFV_basal ganglia	QFV_deep white matter	QFV_cerebellum	QFV_frontal	QFV_insula	QFV_internal capsule	QFV_brainstem	QFV_occipital	QFV_parietal	QFV_temporal	QFV_thalamus
**basal ganglia**	0.011	0.390	0.002	-0.001	-0.002	0.015	0.016	-0.003	-0.002	-0.004	-0.010	-0.003
**deep white matter**	0.000	0.004	0.291	0.004	0.013	0.000	0.004	0.000	-0.010	0.068	-0.005	-0.001
**cerebellum**	0.002	0.001	-0.003	0.416	0.000	-0.001	-0.001	0.001	-0.006	0.004	-0.007	0.005
**frontal**	0.003	0.004	0.027	0.002	0.408	0.009	0.002	0.001	0.004	0.011	0.003	0.002
**insula**	0.003	-0.001	0.003	0.000	0.006	0.426	0.006	0.000	0.000	-0.004	0.004	0.000
**internal capsule**	0.131	-0.035	-0.017	0.000	0.008	-0.015	0.169	-0.008	0.000	-0.015	-0.001	0.000
**brainstem**	0.160	-0.001	-0.001	0.011	0.000	-0.001	0.002	0.416	0.000	-0.001	0.000	0.001
**occipital**	0.023	0.019	0.010	0.037	0.005	0.015	0.008	-0.004	0.268	0.002	0.016	0.028
**parietal**	0.008	0.000	0.003	0.004	0.006	0.000	0.003	0.000	0.090	0.304	0.000	0.005
**temporal**	0.001	0.005	0.001	0.004	0.031	0.002	0.001	0.002	-0.002	0.006	0.328	0.003
**thalamus**	0.003	0.019	0.004	0.020	0.000	0.000	0.053	0.002	0.000	0.000	0.002	0.345

Supplementary Table 2. Feature importance as revealed by the analysis of Mean Decrease in Impurity (MDI) and permutation test of the Random Forest (RF) model for brain structures.

	occurrence	BACC	F1	Precision	Sensitivity	Kappa
ACA	25	0.732	0.545	0.632	0.480	0.523
MCA	260	0.888	0.879	0.981	0.796	0.757
PCA	64	0.867	0.814	0.889	0.750	0.787
cerebellar	59	0.920	0.850	0.836	0.864	0.828
basilar	18	0.843	0.553	0.448	0.722	0.531
Lenticulostriate	139	0.827	0.770	0.839	0.712	0.683
Ch&ThalPerf	70	0.766	0.604	0.609	0.600	0.535
basal ganglia	126	0.868	0.824	0.875	0.778	0.763
deep wm	177	0.796	0.749	0.725	0.774	0.585
cerebellum	59	0.904	0.838	0.845	0.831	0.814
frontal	167	0.866	0.829	0.831	0.826	0.733
insula	84	0.896	0.796	0.742	0.857	0.746
internal capsule	39	0.759	0.386	0.267	0.692	0.301
brainstem	45	0.945	0.863	0.820	0.911	0.848
occipital	65	0.798	0.683	0.745	0.631	0.637
parietal	119	0.826	0.727	0.687	0.773	0.626
temporal	111	0.827	0.740	0.750	0.730	0.659
thalamus	42	0.862	0.711	0.667	0.762	0.680

Supplementary Table 3. Performances of RF models to predict infarct location in the testing set (n = 464), using non-linear brain normalization. The numbers are for comparison to the same procedures, using linear brain normalization, as described in Table 3 of the main manuscript

	occurrences	BACC	F1	Precision	Sensitivity	Kappa
ACA	2	0.745	0.500	0.500	0.500	0.490
MCA	65	0.913	0.947	0.926	0.969	0.843
PCA	6	0.807	0.533	0.444	0.667	0.497
cerebellar	7	0.978	0.778	0.636	1.000	0.757
basilar	4	0.870	0.750	0.750	0.750	0.740
Lenticulostriate	20	0.888	0.750	0.643	0.900	0.674
Ch&ThalPerf	8	0.916	0.737	0.636	0.875	0.710
basal ganglia	29	0.875	0.806	0.758	0.862	0.720
deep wm	54	0.748	0.729	0.833	0.648	0.487
cerebellum	8	0.910	0.700	0.583	0.875	0.668
frontal	48	0.892	0.891	0.849	0.938	0.781
insula	37	0.872	0.829	0.756	0.919	0.713
internal capsule	4	0.578	0.143	0.100	0.250	0.091
brainstem	5	0.874	0.571	0.444	0.800	0.542
occipital	6	0.885	0.588	0.455	0.833	0.554
parietal	35	0.820	0.756	0.660	0.886	0.593
temporal	34	0.790	0.716	0.617	0.853	0.531
thalamus	7	0.978	0.778	0.636	1.000	0.757

Supplementary Table 4. Performances of RF models to predict infarct location in an external testing set (STIR, $n = 100$)

	E1 vs. E2	E1 vs. E3	E2 vs. E3	auto vs. E1	auto vs. E2	auto vs. E3
**arterial**						
ACA	0.56 [0.47 0.63]	0.73 [0.67 0.77]	0.69 [0.62 0.74]	0.66 [0.59 0.71]	0.44 [0.33 0.53]	0.63 [0.56 0.7]
MCA	0.83 [0.79 0.86]	0.86 [0.83 0.88]	0.85 [0.82 0.87]	0.90 [0.88 0.92]	0.84 [0.81 0.86]	0.84 [0.81 0.87]
PCA	0.78 [0.73 0.81]	0.89 [0.87 0.91]	0.73 [0.68 0.78]	0.89 [0.87 0.91]	0.74 [0.69 0.78]	0.83 [0.8 0.86]
cerebellar	0.86 [0.83 0.88]	0.92 [0.9 0.93]	0.85 [0.82 0.88]	0.93 [0.92 0.95]	0.85 [0.82 0.87]	0.92 [0.91 0.94]
basilar	0.53 [0.44 0.61]	0.63 [0.56 0.7]	0.91 [0.89 0.92]	0.71 [0.65 0.76]	0.74 [0.69 0.78]	0.79 [0.75 0.82]
Lat.	0.82 [0.78 0.85]	0.85 [0.82 0.88]	0.82 [0.78 0.85]	0.85 [0.82 0.87]	0.84 [0.8 0.86]	0.85 [0.81 0.87]
Choroidal & Thalamoperf.	0.64 [0.57 0.7]	0.73 [0.67 0.77]	0.78 [0.74 0.82]	0.76 [0.71 0.8]	0.75 [0.7 0.79]	0.76 [0.71 0.8]
**brain**						
basal ganglia	0.83 [0.79 0.85]	0.83 [0.79 0.86]	0.84 [0.81 0.87]	0.86 [0.83 0.88]	0.83 [0.79 0.86]	0.83 [0.79 0.86]
deep white	0.57 [0.49 0.64]	0.72 [0.67 0.77]	0.65 [0.58 0.71]	0.76 [0.71 0.8]	0.67 [0.6 0.72]	0.74 [0.69 0.78]
cerebellum	0.89 [0.86 0.9]	0.92 [0.91 0.94]	0.91 [0.89 0.92]	0.92 [0.9 0.93]	0.86 [0.83 0.88]	0.89 [0.87 0.91]
frontal	0.73 [0.67 0.77]	0.80 [0.76 0.83]	0.81 [0.77 0.84]	0.85 [0.82 0.87]	0.77 [0.72 0.81]	0.81 [0.77 0.84]
insula	0.86 [0.83 0.89]	0.83 [0.8 0.86]	0.87 [0.84 0.89]	0.85 [0.82 0.87]	0.88 [0.85 0.9]	0.85 [0.82 0.87]
internal	0.58 [0.49 0.65]	0.65 [0.58 0.71]	0.65 [0.58 0.71]	0.69 [0.62 0.74]	0.55 [0.46 0.63]	0.68 [0.61 0.73]
midbrain, pons	0.88 [0.86 0.9]	0.91 [0.9 0.93]	0.94 [0.92 0.95]	0.91 [0.89 0.92]	0.88 [0.86 0.9]	0.90 [0.88 0.92]
occipital	0.81 [0.77 0.84]	0.75 [0.69 0.79]	0.83 [0.8 0.86]	0.79 [0.74 0.82]	0.78 [0.74 0.82]	0.70 [0.64 0.75]
parietal	0.72 [0.66 0.77]	0.74 [0.68 0.78]	0.84 [0.8 0.86]	0.78 [0.73 0.82]	0.79 [0.74 0.82]	0.78 [0.74 0.82]
temporal	0.73 [0.67 0.77]	0.77 [0.73 0.81]	0.80 [0.76 0.83]	0.81 [0.77 0.84]	0.76 [0.72 0.8]	0.81 [0.77 0.84]
thalamus	0.86 [0.83 0.88]	0.88 [0.86 0.9]	0.86 [0.83 0.88]	0.84 [0.8 0.86]	0.85 [0.82 0.87]	0.89 [0.86 0.9]
**all regions**						
average (stdev)	0.75 (0.12)	0.8 (0.09)	0.81 (0.08)	0.82 (0.08)	0.77 (0.11)	0.81 (0.08)

Supplementary Table 5. ICCs [95% confidence interval] between pair of human evaluators (E1, E2, E3) and between the RF model and each evaluator, in the testing set (n = 464)

Automatic Radiological Report			
Area of restricted diffusion within the right brain hemisphere, with 166.412 ml, in the territory of middle cerebral artery, and lateral lenticulostriate. The area involves the following brain regions: basal ganglia, deep white matter, frontal lobe, insula, temporal lobe, and possibly parietal lobe. There is no hydrocephalus. The predicted MCA – ASPECTS is 2.			
**Arterial Territories affected**			
ROI	predict	predict probability	QFV(% of injury)
anterior cerebral artery	0	0	0.046
middle cerebral artery	1	0.87	0.354
posterior cerebral artery	0	0.109	0.084
cerebellar artery	0	0.027	0
basilar artery	0	0.151	0.005
Lateral Lenticulostriate	1	0.622	0.597
Choroidal & Thalamoperforating	0	0.133	0.306
**Anatomical structures affected**			
ROI	predict	predict probability	QFV(% of injury)
basal ganglia	1	0.608	0.345
deep white matter	1	0.529	0.763
cerebellum	0	0.013	0
frontal lobe	1	0.567	0.054
insula	1	0.762	0.729
internal capsule	0	0.117	0.776
brainstem	0	0.032	0.064
occipital lobe	0	0.146	0.267
parietal lobe	0	0.473	0.136
temporal lobe	1	0.83	0.63
thalamus	0	0.2	0.255
**Quality control (QC)**			
Registration interior QC index = 0.464, average population QC index = 0.629(+-0.046).			
Registration global QC index = 0.714, average population QC index = 0.759(+-0.036).			

Supplementary Note 1. Example of radiological report outputed by ADS (corresponds to the brain showed in Figure 3)

SUPPLEMENTARY FIGURES

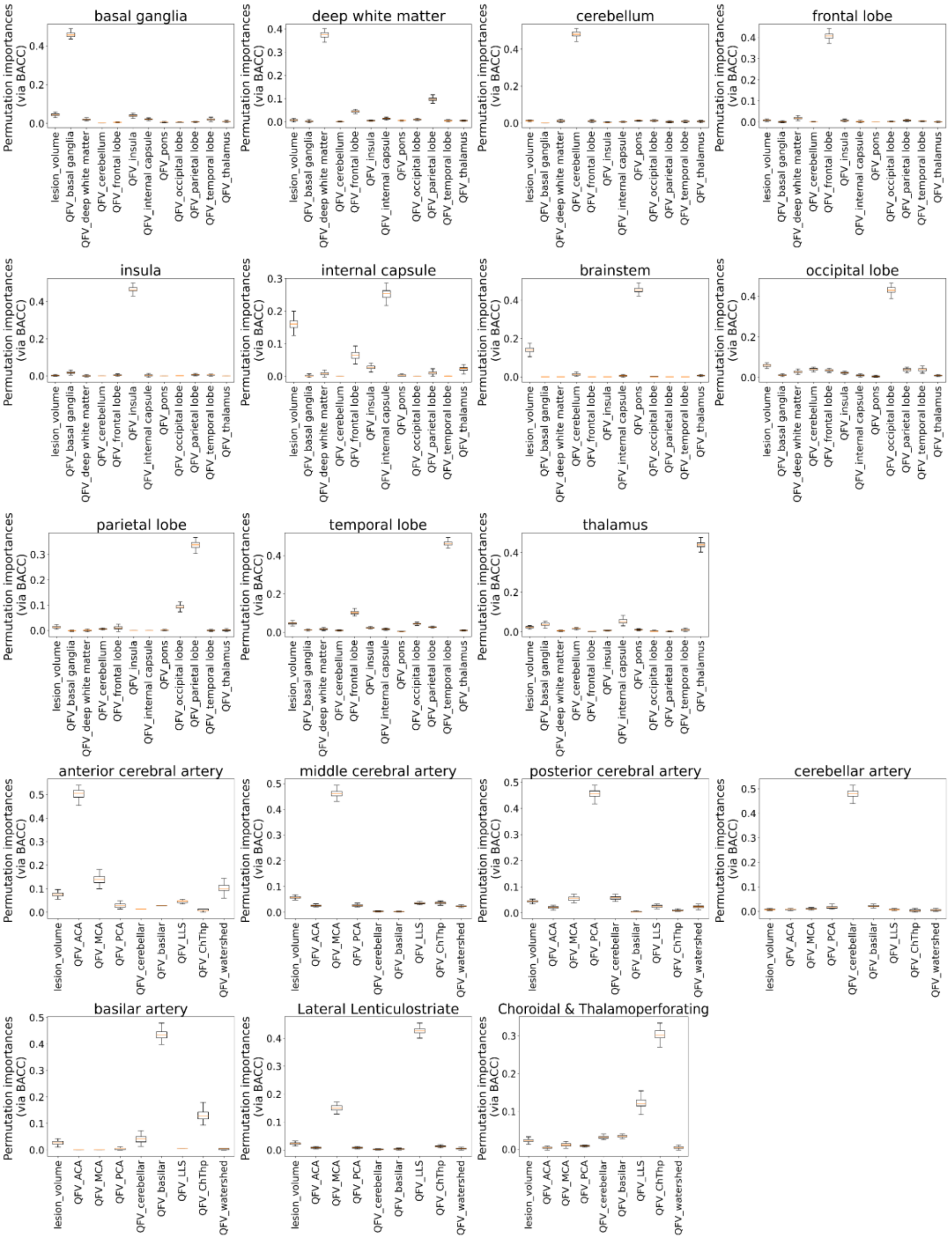

Supplementary Figure 1. Feature importance analysis of the random forest (RF) models accessed by permutation test, in the training set.

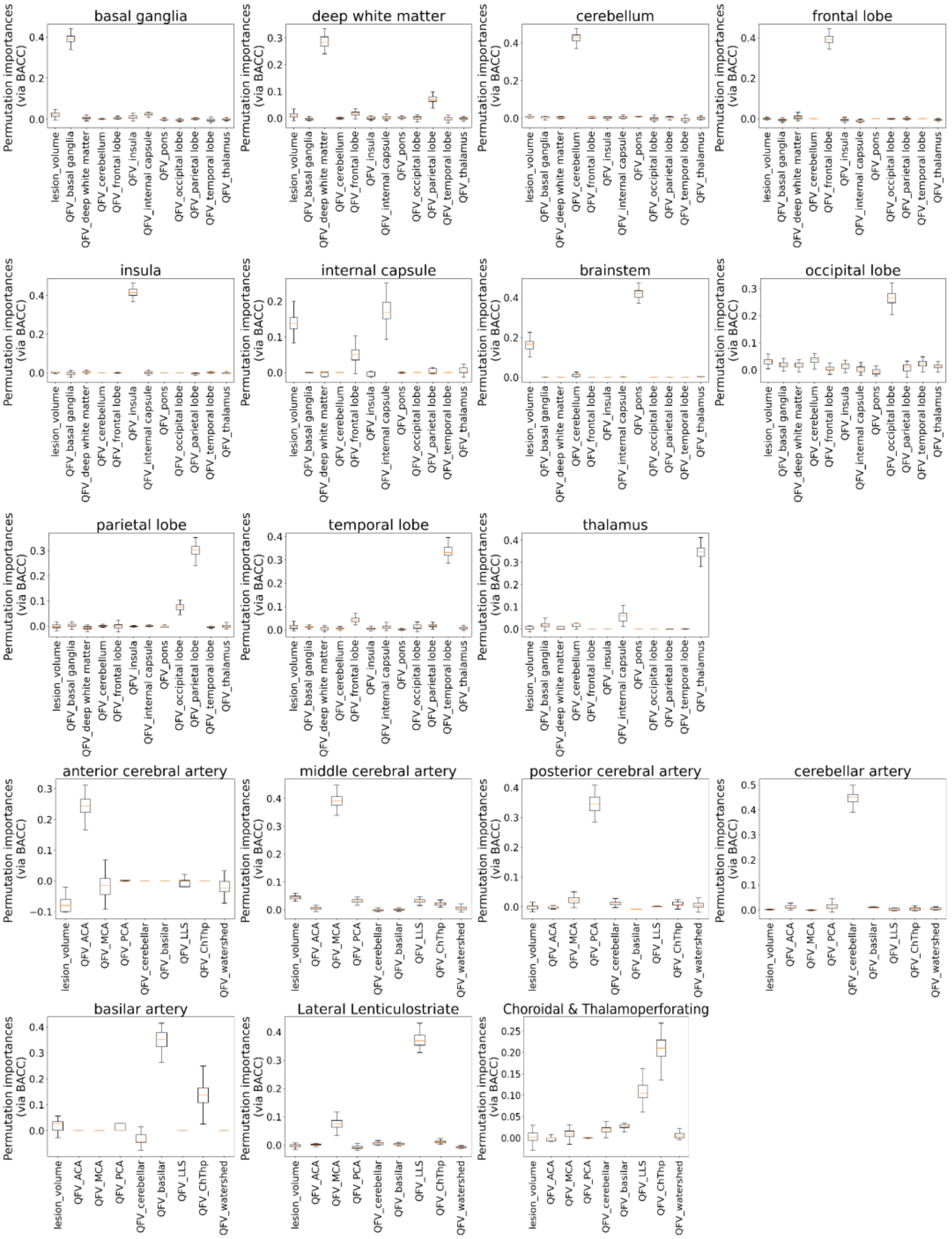

Supplementary Figure 2. Feature importance analysis of the random forest (RF) models accessed by permutation test, in the testing set.

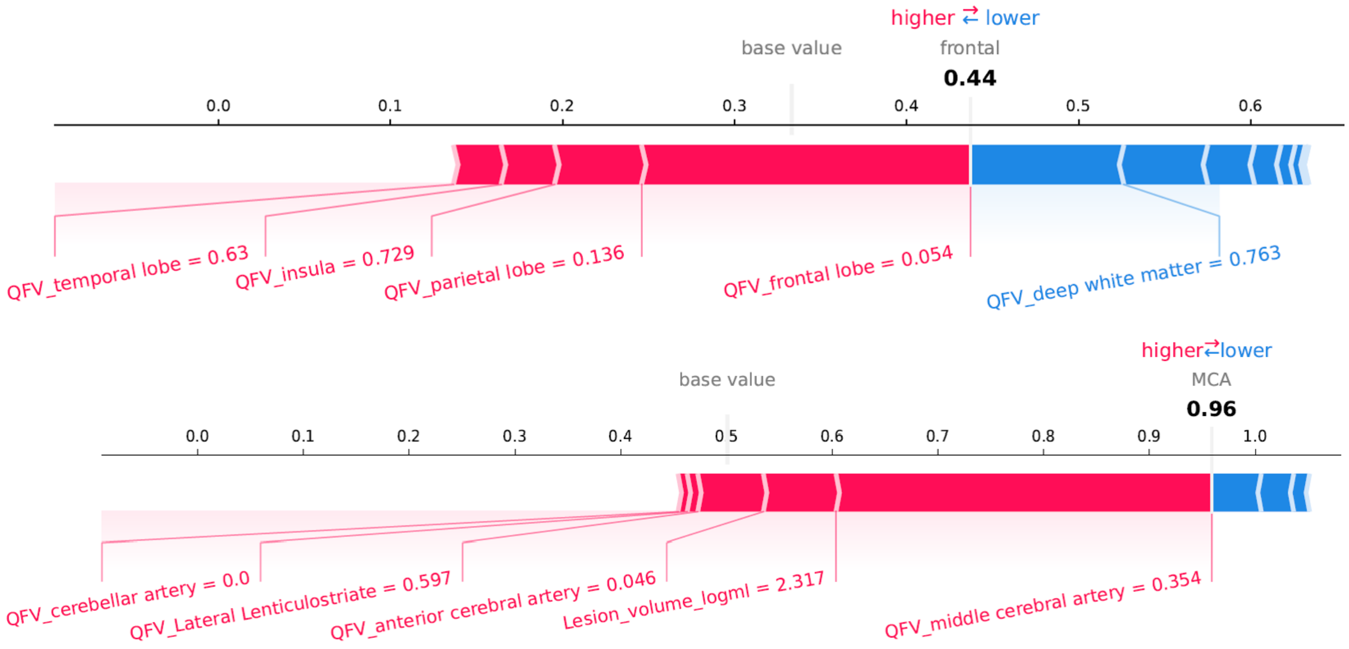

Supplementary Figure 3. Illustrative example of the outputs of our interpretable pre-trained model in ADS. This example (which corresponds to the brain showed in Figure 3) shows how the model explained the contribution of each QFV component to predict infarct in frontal lobe and in the MCA territory, in this case.

SUPPLEMENTARY REFERENCES

1. C. F. Liu, R. Leigh, V. Urrutia, B. Johnson, J. Hsu, X. Xu, S. Mori, A. E. Hillis, and A. V. Faria, "A large dataset of annotated clinical MRIs and linked metadata of patients with acute stroke," under consideration Sci. Data .
2. K. H. Brodersen, C. S. Ong, K. E. Stephan, and J. M. Buhmann, "The balanced accuracy and its posterior distribution," in *2010 20th international conference on pattern recognition,* (IEEE, 2010), pp. 3121–3124.
3. L. Breiman, "Random forests," Mach. learning **45**, 5–32 (2001).
4. M. Abadi, A. Agarwal, P. Barham, E. Brevdo, Z. Chen, C. Citro, G. S. Corrado, A. Davis, J. Dean, M. Devin, S. Ghemawat, I. Goodfellow, A. Harp, G. Irving, M. Isard, Y. Jia, R. Jozefowicz, L. Kaiser, M. Kudlur, J. Levenberg, D. Mané, R. Monga, S. Moore, D. Murray, C. Olah, M. Schuster, J. Shlens, B. Steiner, I. Sutskever, K. Talwar, P. Tucker, V. Vanhoucke, V. Vasudevan, F. Viégas, O. Vinyals, P. Warden, M. Wattenberg, M. Wicke, Y. Yu, and X. Zheng, "TensorFlow: Large-scale machine learning on heterogeneous systems," (2015). Software available from tensorflow.org.
5. F. Chollet *et al.*, "Keras," (2015).
6. "nibabel," doi.org/10.5281/zenodo.41097916 (2020).
7. P. Virtanen, R. Gommers, T. E. Oliphant, M. Haberland, T. Reddy, D. Cournapeau, E. Burovski, P. Peterson, W. Weckesser, J. Bright, S. J. van der Walt, M. Brett, J. Wilson, K. J. Millman, N. Mayorov, A. R. J. Nelson, E. Jones, R. Kern, E. Larson, C. J. Carey, İ. Polat, Y. Feng, E. W. Moore, J. VanderPlas, D. Laxalde, J. Perktold, R. Cimrman, I. Henriksen, E. A. Quintero, C. R. Harris, A. M. Archibald, A. H. Ribeiro, F. Pedregosa, P. van Mulbregt, and SciPy 1.0 Contributors, "SciPy 1.0: Fundamental Algorithms for Scientific Computing in Python," Nat. Methods **17**, 261–272 (2020).
8. E. Garyfallidis, M. Brett, B. Amirbekian, A. Rokem, S. Van Der Walt, M. Descoteaux, and I. Nimmo-Smith, "Dipy, a library for the analysis of diffusion mri data," Front. neuroinformatics **8**, 8 (2014).
9. S. Van der Walt, J. L. Schönberger, J. Nunez-Iglesias, F. Boulogne, J. D. Warner, N. Yager, E. Gouillart, and T. Yu, "scikit-image: image processing in python," PeerJ **2**, e453 (2014).
10. F. Pedregosa, G. Varoquaux, A. Gramfort, V. Michel, B. Thirion, O. Grisel, M. Blondel, P. Prettenhofer, R. Weiss, V. Dubourg *et al.*, "Scikit-learn: Machine learning in python," J. machine learning research **12**, 2825–2830 (2011).

11. S. M. Lundberg and S.-I. Lee, “A unified approach to interpreting model predictions,” Adv. neural information processing systems **30** (2017).
