# Supplementary material for: Automatic comprehensive radiological reports for clinical acute stroke MRIs
Source: Commun Med (Lond). 2023 Jul 10;3:95. doi: 10.1038/s43856-023-00327-4 (PMC10333348; doi:10.1038/s43856-023-00327-4)
Supplement: Supplementary file 10 — Reporting Summary [file 43856_2023_327_MOESM10_ESM.pdf]

nature portfolio

Corresponding author(s): Andreia V Faria

Last updated by author(s): 2023/05/17

Reporting Summary

Nature Portfolio wishes to improve the reproducibility of the work that we publish. This form provides structure for consistency and transparency in reporting. For further information on Nature Portfolio policies, see our Editorial Policies and the Editorial Policy Checklist.

Statistics

For all statistical analyses, confirm that the following items are present in the figure legend, table legend, main text, or Methods section.

n/a	Confirmed	
☐	☒	The exact sample size (*n*) for each experimental group/condition, given as a discrete number and unit of measurement
☐	☒	A statement on whether measurements were taken from distinct samples or whether the same sample was measured repeatedly
☐	☒	The statistical test(s) used AND whether they are one- or two-sided<br>*Only common tests should be described solely by name; describe more complex techniques in the Methods section.*
☐	☒	A description of all covariates tested
☒	☐	A description of any assumptions or corrections, such as tests of normality and adjustment for multiple comparisons
☐	☒	A full description of the statistical parameters including central tendency (e.g. means) or other basic estimates (e.g. regression coefficient) AND variation (e.g. standard deviation) or associated estimates of uncertainty (e.g. confidence intervals)
☒	☐	For null hypothesis testing, the test statistic (e.g. *F*, *t*, *r*) with confidence intervals, effect sizes, degrees of freedom and *P* value noted<br>*Give P values as exact values whenever suitable.*
☒	☐	For Bayesian analysis, information on the choice of priors and Markov chain Monte Carlo settings
☒	☐	For hierarchical and complex designs, identification of the appropriate level for tests and full reporting of outcomes
☒	☐	Estimates of effect sizes (e.g. Cohen's *d*, Pearson's *r*), indicating how they were calculated

Our web collection on statistics for biologists contains articles on many of the points above.

Software and code

Policy information about availability of computer code

Data collection	n/a
Data analysis	All codes are publicly available at https://www.nitrc.org/projects/ads and https://zenodo.org/record/5565230, DOI 10.5281/zenodo.5565230

For manuscripts utilizing custom algorithms or software that are central to the research but not yet described in published literature, software must be made available to editors and reviewers. We strongly encourage code deposition in a community repository (e.g. GitHub). See the Nature Portfolio guidelines for submitting code & software for further information.

Data

Policy information about availability of data

All manuscripts must include a data availability statement. This statement should provide the following information, where applicable:

- Accession codes, unique identifiers, or web links for publicly available datasets
- A description of any restrictions on data availability
- For clinical datasets or third party data, please ensure that the statement adheres to our policy

The data used in this study are available at https://www.icpsr.umich.edu/web/ICPSR/studies/38464. These data is public and free and can be downloaded directly from this repository after signing the Disclosure of User Agreement.

Research involving human participants, their data, or biological material

Policy information about studies with human participants or human data. See also policy information about sex, gender (identity/presentation), and sexual orientation and race, ethnicity and racism.

Reporting on sex and gender	We included 1,878 mutually exclusive MRIs with evidence of ischemic stroke in the diffusion weighted images (DWI). The flowchart for data inclusion and demographics is shown in Figure 1. We used data from a National Stroke Center. These data originated from multiple hospitals and a large geographic region, reflecting the profile of the national population with stroke
Reporting on race, ethnicity, or other socially relevant groupings	We included 1,878 mutually exclusive MRIs with evidence of ischemic stroke in the diffusion weighted images (DWI). The flowchart for data inclusion and demographics is shown in Figure 1. We used data from a National Stroke Center. These data originated from multiple hospitals and a large geographic region, reflecting the profile of the national population with stroke
Population characteristics	This is a subset of the "Annotated Clinical MRIs and Linked Metadata of Patients with Acute Stroke", an anonymized dataset organized under waiver of patient consent (IRB00228775), publicly shared at https://doi.org/10.3886/ICPSR38464.v5. The documentation and data dictionary available in the link describes all the population demographic and clinical characteristics
Recruitment	This study included MRIs of patients admitted to the Comprehensive Stroke Center at Johns Hopkins Hospital with the clinical diagnosis of ischemic stroke, between 2009 and 2019. We used data from a National Stroke Center. These data originated from multiple hospitals and a large geographic region, reflecting the profile of the national population with stroke
Ethics oversight	This is a subset of the "Annotated Clinical MRIs and Linked Metadata of Patients with Acute Stroke", an anonymized dataset organized under waiver of patient consent (IRB00228775), publicly shared. We have complied with all relevant ethical regulations from the Johns Hopkins Institutional Review Board that approved this study (IRB00290649).

Note that full information on the approval of the study protocol must also be provided in the manuscript.

Field-specific reporting

Please select the one below that is the best fit for your research. If you are not sure, read the appropriate sections before making your selection.

☒ Life sciences ☐ Behavioural & social sciences ☐ Ecological, evolutionary & environmental sciences

For a reference copy of the document with all sections, see nature.com/documents/nr-reporting-summary-flat.pdf

Life sciences study design

All studies must disclose on these points even when the disclosure is negative.

Sample size	ok
Data exclusions	ok
Replication	ok
Randomization	ok
Blinding	ok

Reporting for specific materials, systems and methods

We require information from authors about some types of materials, experimental systems and methods used in many studies. Here, indicate whether each material, system or method listed is relevant to your study. If you are not sure if a list item applies to your research, read the appropriate section before selecting a response.

Materials & experimental systems

n/a	Involved in the study	
☒	☐	Antibodies
☒	☐	Eukaryotic cell lines
☒	☐	Palaeontology and archaeology
☒	☐	Animals and other organisms
☐	☒	Clinical data
☒	☐	Dual use research of concern
☒	☐	Plants

Methods

n/a	Involved in the study	
☒	☐	ChIP-seq
☒	☐	Flow cytometry
☐	☒	MRI-based neuroimaging

Clinical data

Policy information about clinical studies

All manuscripts should comply with the ICMJE guidelines for publication of clinical research and a completed CONSORT checklist must be included with all submissions.

Clinical trial registration	not a clinical trial, this is a retrospective analysis of anonymized data (now public) organized under a waiver of consent
Study protocol	IRB00290649
Data collection	from public dataset, organized under waiver of patient consent (IRB00228775)
Outcomes	comparison of automated reports with human reports generated for research

Magnetic resonance imaging

Experimental design

Design type	not functional MRIs, but clinical diffusion weighted MRIs
Design specifications	retrospective, cross-sectional
Behavioral performance measures	n/a

Acquisition

Imaging type(s)	diffusion (not acquired for the study)
Field strength	1.5 and 3T
Sequence & imaging parameters	variable, collected over 10 years in multiple scanners as defined in the manuscript
Area of acquisition	brain

Diffusion MRI ☒ Used ☐ Not used

Parameters: clinical DWI protocols, collected over 10 years in multiple scanners, as defined in the manuscript

Preprocessing

Preprocessing software	developed in this study, now public, as defined in the manuscript
Normalization	linear and non-linear as defined in the manuscript
Normalization template	MNI, as defined in the manuscript
Noise and artifact removal	n/a
Volume censoring	manual, defined in the manuscript

Statistical modeling & inference

Model type and settings	unsupervised machine learning, as defined in the manuscript
Effect(s) tested	n/a

Specify type of analysis: ☐ Whole brain ☒ ROI-based ☐ Both

Anatomical location(s): atlases developed for this study are defined in the manuscript

Statistic type for inference (See Eklund et al. 2016)	n/a
Correction	n/a

Models & analysis

n/a	Involved in the study
☒	☐ Functional and/or effective connectivity
☒	☐ Graph analysis
☐	☒ Multivariate modeling or predictive analysis

Multivariate modeling and predictive analysis: All defined in the Methods (main results based in random forest)
